# Relationships of Cardiorespiratory Fitness with Metabolic Risk Factors, Inflammation, and Liver Transaminases in Overweight Youths

**DOI:** 10.1155/2010/580897

**Published:** 2010-06-28

**Authors:** Dominique Bouglé, Gautier Zunquin, Bruno Sesbouë, Jean-Pierre Sabatier

**Affiliations:** ^1^Service de Pédiatrie, Centre Hospitalier de Bayeux, 14400 Bayeux, France; ^2^Service de Pédiatrie, CHU de Caen, 14000, Caen, France; ^3^Institut Régional de Médecine du Sport, CHU de Caen, 14000, Caen, France

## Abstract

The aim of this study was to assess the relationships of fatness and fitness with metabolic risk factors, including liver transaminases and inflammation in obese youth, taking in account gender, age, and pubertal stage. 241 children were studied (135 girls), age 11.9 ± 2.2 years (*x* ± SD), Body Mass Index *z* score 5.4 ± 2.7. For girls, VO_2max_ was significantly associated with insulin (*P* = .001), Insulin resistance (HOMA-IR) (*P* = .005), and ALT (*P* = .012); a relationship was displayed between fibrinogen and age and % fat mass (FM) (*P* = .008); for boys, relationships were found between VO_2max_ and diastolic blood pressure and triglycerides; independent associations were also found between age and insulin, HOMA-IR and HDL cholesterol; fibrinogen and sedimentation rate were related (*P* ≤ .004) with %FM. Their relationships are observed from young age and increase with the continuous increase of factors. This supports the need to treat overweight as soon as it is detected; improving CRF is one of the ways which could be used to prevent the complications of obesity.

## 1. Introduction

Overweight is associated with an increased cardiovascular risk, even in youth [1, 2]. It is also associated with a decreased cardiorespiratory fitness (CRF), which is liable to contribute to obesity itself. Several large reports clearly show a relationship between decreased CRF and the occurrence of metabolic risk factors in overweight adults and youth. Whether fitness and fatness have independent influences on metabolic risk, however, is not fully explained [3, 4].

Some of the studies addressed only one individual factor [5–9] but most of them clustered these risks in “metabolic syndrome” (MS) [8, 10–13]; MS usually associates with obesity, dyslipoproteinemia (raised triglyceride and/or reduced HDL-cholesterol levels), hypertension, and insulin resistance or diabetes, but variables included in the MS and their relative weight vary among definitions; the last of them was given by the International Diabetes Federation [14]. Nonalcoholic fatty liver disease is frequently associated with MS, so that it has been proposed as a core feature of it [15, 16]. In addition inflammation is never included in the criteria of MS, while it seems to be involved in the development of its consequences [1, 17].

The value of MS concept itself is still debated [18]. A recent WHO Expert Consultation came to the conclusions that it is not a useful diagnostic or management tool [19]. 

The present study assessed the respective relationships of CRF and fatness on individual components of MS, including liver transaminases as a surrogate of nonalcoholic fatty liver disease [15, 16]; fibrinogen and sedimentation rate were added as markers of inflammation; CRF was measured by determining the maximal oxygen consumption (VO_2max_); the marker of overweight was fat mass, instead of BMI, because this latter one is a less valid measurement of body fatness in children and does not discriminate between muscle and fat [20] and it has been shown to be less discriminant than fatness as suggested by a recent report [21]; we took into account the gender and the pubertal development of adolescents, owing to the changes that occur in their lifestyle and hormonal status.

## 2. Subjects and Methods

The study is part of a research protocol on the genetics of child obesity, which has been approved by the Ethics Committee of the Hôtel Dieu Hospital (Paris); a written consent to the inclusion in the study was given by each child's parents and by adolescents themselves [22]. 

Subjects were children and adolescents attending the specialized clinic of University Hospital of Caen; they were routinely screened before treatment. 

The clinical examination of children was performed by the same clinician. They were all apparently healthy with no obvious endocrine disease. Pubertal stage was assessed according to Tanner criteria [23]. Weight was assessed with an electronic beam balance; height was measured twice by the same examiner to the nearest 0.5 cm. Body mass index (BMI) was calculated (kg/m²). Patients were considered as overweight when their BMI was over 2 SD of the available French charts [24], which fit better with the local population than the IOTF charts; data were also compared with IOTF cut off points [25]. Blood pressure (BP) was measured by an automatic device on a sitting patient, at least twice, at the end of a consultation (Agilent A1; Agilent 91745 Massy France). 

Blood sample was drawn after an overnight fast, for measurements of plasma glucose, insulin, triglycerides, HDL and LDL cholesterol, alanine aminotransferase (ALT), aspartate aminotransferase (AST), sedimentation rate and fibrinogen. Homeostasis model assessment-insulin resistance (HOMA-IR) was calculated as a measure of insulin resistance (insulin (mU/L) ∗ plasma glucose (mmol/L)/22.5). 

Body composition (total and relative (%) fat and fat-free mass) was measured by X-ray absorptiometry (DEXA) (HOLOGIC QDR-4500 A) [26].

VO²Max was measured by a previously validated protocol reported [26]; it was adapted from Achten [27]; the use of this graded exercise to exhaustion gives an exact value of the peak VO² [26, 27]; subjects performed a graded exercise on a bicycle ergometer (ERGOLINE 500, Bosch) linked to a gas analyzer (Ergocard SCHILLER). Before the test, the children were given several minutes to familiarise themselves with the ergometer and to adapt to the valve/mouthpiece. The subjects warmed up for 5 minutes at 0 W. The first step was fixed at 30 W with a rectangular progression of 20 W every 3 minutes 30 seconds, until exhaustion. 

### 2.1. Statistical Analysis

Results were expressed as mean ± 1 SD; statistical analysis used univariate regression analysis between VO_2max_ and anthropometric and biologic data; multivariate analyses performed on each gender; they used age and % fat mass as covariates of VO_2max_. Tanner stage was also tested as covariate.

The level of significance was set at *P* < .05.

## 3. Results

241 children were studied (135 girls), aged 11.9 ± 2.2 years (*x* ± SD), range: 6.2 ± 17.9; their BMI *z* score was 5.4 ± 2.7. 

Descriptive data are given in Table 1. 

Univariate regressions (Table 2) displayed significant relationships between VO_2max_ and age, pubertal development, BMI, body composition parameters, insulin and HOMA-IR, lipids and ALT (≤0.02), and inflammation parameters (*P* ≤ .0005). 

Table 3 shows the results of multivariate regression analysis. Since Tanner stage did not enter in any model, it was discarded from analysis. For girls, VO_2max _ remained significantly associated with insulin (*P* = .001), HOMA-IR (*P* = .005), and ALT (*P* = .012); a relationship was displayed between fibrinogen and age and % fat mass (*P* = .008); for boys, relationships were found between VO_2max_ and diastolic BP and triglycerides; independent associations were also found between age and insulin, HOMA-IR, and HDL cholesterol; fibrinogen and sedimentation rate were related (*P* ≤ .004) with %FM.

## 4. Discussion

CRF and adiposity are associated with metabolic risk factors and cardiac and vascular risks; it is known that these risks are already present in youth [2, 28]. The main roles are attributed either to fitness or fatness [3, 8]. In fact an influence of both variables could be expected owing to the relationships between obesity and movement efficiency [29] which lately led to social exclusion, but differences in statistical analysis, ways of assessing metabolic risk, adiposity, and/or taking account of gender and sexual development could explain at least part of these discrepancies. 

The potential metabolic interdependence between these risks lead to the concept of metabolic syndrome. Currently this concept is debated [18, 19], and these risk factors could have only statistical relations. So studying the individual relationships between each of them and with fitness or adiposity could be of interest to understand the mechanisms involved in the health risks of obesity; it could also give more specific aims to improve the efficiency of prevention campaigns [30]. 

The present study showed mainly a relation between CRF (VO_2max_) and insulin metabolism in girls; VO_2max_ was significantly related to diastolic BP in boys and to ALT in girls. Age was mainly involved in the relationships with insulin and lipids in boys and with inflammation in girls. Apart from the relationship between inflammation parameters in boys, the degree of adiposity did not appear to play a main influence on the early occurrence of risk factors. None of these relationships depended on pubertal development. 

It seemed useful to address current adiposity and not only BMI which is the sum of fat free and fat mass [20]; a previous study shows that measured fat mass is a better variable to assess the metabolic risk of obesity, owing to the endocrine and inflammatory actions of adipose tissue [1, 21]; most of the studies use BMI as marker of adiposity [13]; their conclusions could be taken with caution since an increased BMI can be associated with an increased muscle mass, specifically in active boys.

Subjects of the present study did not fulfil the criteria of metabolic syndrome, whatever the definition used; it is known that the risk of developing MS factors increases with the degree of overweight [1, 10, 18]. In some reports, the relationships between MS and CRF only occur at high degrees of adiposity [9]. Our regression analysis showed a direct effect of age on inflammation and lipoproteins, from young age and from a moderate degree of excess adiposity; these observations, added to the early development of arterial dysfunction [28] and the relationship between child obesity and its complications in adult [31], support a precocious treatment of overweight in youth.

When it is assessed, no effect of pubertal status is observed [9].

Differences were found between boys and girls in the relationships between risk factors (lipid profile, insulin resistance) and fitness by some authors [7, 32, 33] and not by others [9]; such differences were expected, due to the difference in insulin sensitivity between both sexes [34]. They should not result from sex hormones, since no effect of pubertal stage was found; more differences are found in lifestyle; boys are more active than girls, and physical activity affects all cardiovascular risks [7, 17].

Imperatore finds a significant association between insulin sensitivity and CRF only in boys [7]; however, since only BMI was measured, this association could have resulted from a higher muscle mass in boys rather than from an increased adiposity; the relationship between fasting glucose and CRF disappears when body fat is taken into account; in the same report, HOMA and fasting glucose are negatively associated with CRF only in the higher body fat tertile [9]; Gutin finds also a relationship between VO_2max_ and the percentage of body fat mass [21, 32] while others find no relationship between VO_2max_ and insulin sensitivity [11]. CRF could interact with metabolic risk factors through insulin resistance. Insulin resistance is reported as the key mechanism in the development of metabolic complications of obesity [1], yet these models usually do not take physical activity or CRF into account. Physical training enhances insulin sensitivity in the exercised muscle and enhances muscle contraction-induced glucose uptake in the muscle; the mechanisms include postreceptor insulin signalling, increased glycogen synthesis, and enhanced muscle capillarization and blood flow [35]. 

Obesity is a low-grade inflammation disease, positively associated with body fat and negatively with CRF [9]; this inflammation, yet not included in MS criteria, is a possible pathway in its pathogenesis: it induces insulin resistance and MS; insulin resistance promotes inflammation further through an increase in free fatty acids [17].

The increase in liver transaminases is associated to other components of MS, particularly to insulin resistance [15, 16, 33, 36]; the precise relationship between fatty liver and MS is not precisely known but is likely to also involve insulin resistance [37, 38]; in addition, the present study shows that it is under regulation of CRF, such as other risk factors [38]. 

So far, no specific treatment of nonalcoholic fatty disease has been proposed [39]; it is known that weight loss improves liver dysfunction [32, 33]; the present observation suggests that lifestyle interventions aimed at enhancing CRF should also be efficient in improving liver parameters.

## 5. Conclusions

CRF is one of the parameters associated with individual metabolic risk factors in young overweight subjects, together with age and percentage of fat mass. As expected, the main relationship was found with insulin resistance. CRF was also associated with other variables often associated with metabolic risk of obesity, that is, transaminases and inflammation. 

The relationships differed between boys and girls and were not influenced by their pubertal stage. They were observed from young age and increased continuously with the factors studied.

This supports the need to treat overweight as soon as it is detected; improving CRF is one of the ways which could be used to prevent the complications of obesity.

## Figures and Tables

**Table 1:** Descriptive data.

		Girls		Boys	
	Unit	*X*	*SD*	*x*	*SD*
*N*		135		106	
Age	Years	11.9	2.3	12.1	2.3
Tanner		3.0	1.8	2.0	1.3
Weight	k	62.4	16.8	69.0	21.2
Height	m	1.52	0.11	1.55	0.12
BMI	k/m²	26.7	4.5	28.1	5.8
*z* Score BMI		5.1	2.5	5.8	2.9
Overweight (IOTF cut off points)	%	47		37	
Obesity (IOTF cut off points)	%	53		63	
Fat Free Mass	%	56.6	6.4	57.6	6.8
Fat Mass	%	40.6	6.4	39.3	6.6
VO_2max _	mL O²/k/min	25.5	5.1	27.9	6.1
Systolic BP	MmHg	116	19	120	13
Diastolic BP	MmHg	61	12	65	10
Fasting Glucose	mmol/L	4.7	0.4	4.9	0.4
Fasting Insulin	mU/L	10.4	5.4	10.9	7.2
HOMA-IR		2.1	1.1	2.4	1.7
HDL Cholesterol	Mmol/L	1.5	0.4	1.4	0.3
LDL Cholesterol	mmol/L	2.8	0.9	2.7	1.3
Triglycerides	mmol/L	0.8	0.4	0.9	0.5
AST	IU/L	23	8	24	7
ALT	IU/L	24	7	28	17
Sedimentation rate		13	6	12	7
Fibrinogen	g/L	3.6	0.8	3.6	0.7

**Table 2:** Univariate regression analysis between VO_2max_ and metabolic risk factors.

	*P*	*F*	*T*
Age	.0062	7,64	−2.976
Tanner stage	.0018	10.043	−3.169
BMI	<.0001	83.809	−9.55
*z* score	<.0001	48.949	−6.99
Fat Free Mass kg	.0002	14.576	−3.818
Fat Free Mass %	<.0001	43.856	6.622
Fat Mass kg	<.0001	92.285	−9.761
Fat Mass %	<.0001	56.838	−7.539
Systolic BP	.124		
Diastolic BP	.072		
Fasting Glucose	.220		
Fasting Insulin	<.0001		−5.52
HOMA-IR	<.0001		−4.98
Triglycerides	.010		−2.59
HDL Cholesterol	.019		2.36
LDL Cholesterol	.509		
AST	.770		
ALT	.0007		−3.421
Sedimentation rate	.0002	14.26	−3.776
Fibrinogen	.0005	8.012	−2.831

**Table 3:** Multivariate regression between metabolic risk factors and VO²Max, using age and % fat mass as covariates.

	Girls			Boys		
	F	*P*		F	*P*	
Systolic BP	1.938	.129		2.508	.066	
Diastolic BP	0.201	.896		3.294	.025	VO²Max: 0.048
Fasting Glucose	1.476	.226		1.968	.125	
Fasting Insulin	5.683	.001	VO²Max: 0.002	6.317	<.0001	Age: 0.017
HOMA-IR	4.530	.005	VO²Max: 0.001	6.275	<.0001	Age: 0.026
Triglycerides	2.384	.073		3.976	.010	VO²Max: 0.034
HDL Cholesterol	1.055	.371		3.139	.029	Age: 0.039
LDL Cholesterol	0.846	.472		0.930	.431	
AST	1.051	.373		0.514	.674	
ALT	3.829	.012	VO²Max: 0.073	2.492	.065	
Fibrinogen	4.21	.008	Age: 0.040 % FM: 0.007	2.938	.038	%FM: 0.018
Sedimentation rate	4.077	.009		4.085	<.001	%FM: 0.045

Tanner stage did not significantly enter in any model tested.
